# Case Report: Ophthalmologic Evaluation Over a Long Follow-Up Time in a Patient With Wolfram Syndrome Type 2: Slowly Progressive Optic Neuropathy as a Possible Clinical Finding

**DOI:** 10.3389/fped.2021.661434

**Published:** 2021-04-29

**Authors:** Valentina Di Iorio, Enza Mozzillo, Francesco Maria Rosanio, Francesca Di Candia, Rita Genesio, Francesco Testa, Claudio Iovino, Adriana Franzese, Francesca Simonelli

**Affiliations:** ^1^Eye Clinic, Multidisciplinary Department of Medical, Surgical and Dental Sciences, Università degli Studi della Campania ‘Luigi Vanvitelli’, Naples, Italy; ^2^Section of Pediatrics, Department of Translational Medical Science, Regional Center of Pediatric Diabetes, Federico II University of Naples, Naples, Italy; ^3^Department of Molecular Medicine and Medical Biotechnology, University of Naples, Naples, Italy

**Keywords:** CISD2 gene, optic atrophy, optic neuropathy, neurodegeneration, non-autoimmune diabetes, Wolfram syndrome

## Abstract

Wolfram syndrome (WFS) is a rare autosomal recessive neurodegenerative disease whose diagnosis requires diabetes mellitus and optic atrophy (OA). WFS includes a wide spectrum of other possible complications such as diabetes insipidus, sensorineural deafness, urinary tract problems, neurological and psychiatric disorders. Most WFS patients show type 1 syndrome (WFS1) caused by mutations in the WFS1 gene, encoding Wolframin protein, while few patients are affected by WFS type 2 (WFS2) due to a pathogenetic variants in the CISD2 gene encoding an endoplasmic reticulum intermembrane small protein. WFS2 is considered a phenotypic and genotypic variant of WFS, from which differs only for the increased risk of bleeding and presence of peptic ulcers. OA and diabetes are considered cardinal features of WFS. We hereby report the ophthalmologic evaluation in a patient, previously described, with WFS2 after 8 years of follow-up. A 20-year-old white woman was referred to our retinal center for the first time in 2012 following a diagnosis of a novel intragenic exon 2 CISD2 homozygous deletion, for the suspicion of an associated bilateral OA. Fundus examination, spectral-domain optical coherence tomography, visual field, visual evoked potentials were performed and confirmed the presence of an optic neuropathy that remained stable over 8 years follow up. A slowly progressive optic neuropathy, rather than OA can characterize patients with WFS2 and CISD2 intragenic deletion.

## Introduction

Wolfram syndrome (WFS) is a rare autosomal recessive neurodegenerative disease characterized by a wide phenotypic spectrum including non-autoimmune diabetes mellitus (DM) (1), and optic atrophy (OA), often associated with diabetes insipidus (DI) and deafness, hence the historical acronym: DIDMOAD (2). Other common clinical signs include urinary tract problems and renal dysfunction, related to neurogenic bladder, endocrine disorders, severe gastrointestinal ulcers, psychiatric symptoms, and progressive neurological degeneration (2). The most frequent form of WFS type 1 (WFS1) includes mutations in the WFS1 gene, whereas the rare form of WFS type 2 (WFS2) involves the CISD2 gene. There is no clear genotype-phenotype correlation in this complex syndrome (3). WFS2 is distinguishable from WFS1 by the increased risk of bleeding, presence of peptic ulcers and the absence of DI, albeit a case of a patient with WFS2 and DI has been reported (4). WFS2 (MIM #604928) was firstly described in the year 2000 in a large consanguineous Jordanian family (5). Only in 2007 it was reported to be linked to a missense mutation in a novel gene, CISD2, mapping in 4q24 (6). The CISD2-encoded endoplasmic reticulum intermembrane small protein, does not interact directly with Wolframin, the WFS1 gene-encoded protein (6).

Our group reported the first Italian Caucasian girl with WFS2 (7), carrying a novel intragenic exon 2 CISD2 homozygous deletion from C-4OKFJ (102,885,416 bp) to C-6MGSK (102,886,154 bp), mapping on chromosome 4q24, showing optic neuropathy rather than OA, and impaired platelet aggregation to adenosine diphosphate and not to collagen, as described in the Jordanian family (5). Since this mutation had never been reported, consanguinity was hypothesized. Although patient's parents reported to be not consanguineous a microsatellite analysis confirmed the hypothesis of a common ancestor (7).

Later on, some authors reported other two Italian siblings with a novel homozygous CISD2 mutation mapping within the donor splice site of intron 1. Authors hypothesized that the alteration presumably would have caused a skipping of exon 1 with subsequent disrupting of the mRNA splicing by eliminating exon 2 (4). Among the clinical peculiar features of WFS, the two sisters presented both DM, deafness and peptic ulcers. The platelet aggregation defect was present only in the youngest sister, while the eldest showed DI, not diagnosed with the water deprivation test. OA was described in both patients (4). OA is considered one of the cardinal features of WFS and generally occurs after onset of DM at an average age of 11 years with a progressive loss of visual acuity and color vision (2). In fact, clinical suspicion of WFS is mainly based on the observation of OA after the diagnosis of diabetes in patients. Although there are currently no effective therapies for OA, annual eye examination is highly recommended. Retinal thinning is also considered a reliable marker of disease progression (8).

We hereby report the evolution of optic neuropathy over 8 years of follow-up of our patient with WFS2 and CISD2 intragenic deletion.

## Case Presentation

The previous clinical history of our patient, who currently is a young woman of 28 years, has been described in the previous report (7). She came to our attention when she was 18 years old presenting non-autoimmune insulin-dependent diabetes mellitus, sensorineural hearing loss, intestinal ulcers, optic neuropathy, and defective platelet aggregation to ADP.

Actually, her diabetes is moderately-controlled (latest glycated hemoglobin values 7%) with a very low total daily dose of insulin requirement (<0.5 IU/kg).

The annual audiological evaluation showed a stable mild bilateral sensorineural hearing loss on high frequencies, without the need for hearing aids throughout the follow-up period.

She is still on pump inhibitor therapy and no longer presented peptic ulcers at endoscopic controls.

From July 2014, medical history was characterized by the presence of recurrent febrile urinary tract infections. Renal ultrasonography was performed showing grade IV vesicoureteral reflux (VUR), for which prophylactic antibiotic therapy was started, and bilateral pyelectasis with thickened distended bladder. Voiding cystourethrography and urodynamic test confirmed severe VUR and showed a low-capacity corrugated-walled bladder with sphincteric dyssynergia and pathological post-voiding residue. Diagnosis of neurogenic bladder was made, and the attempt of oxybutynin therapy failed due to drug side effects (blurred and flashing vision). Intermittent bladder catheterization was started. Patient underwent annual endoscopic examinations for peptic ulcers, which did not recur, and had no further bleeding episodes. Apart from the neurogenic bladder, our patient did not develop any other neurological disorders. Currently, she has a successful career as a lawyer.

In March 2020, she performed the last brain MRI that showed a slight increase in the antero-posterior diameter of both eyes with mild prominence of the CSF sheaths of both optic nerves, mild lower worm hypoplasia and multiple T2/FLAIR hyperintense areas in the cerebral bi-hemispheric white matter.

From 2012 to date, she has been regularly followed up at the Referral Center for Inherited Retinal Dystrophies of the Eye Clinic, University of Campania “Luigi Vanvitelli.” Periodic ophthalmological examinations were performed, including best corrected visual acuity (BCVA), measurements with the Snellen visual chart, slit lamp anterior segment examination, measurements of intraocular pressure, fundus examination, Octopus visual fields assessment (Octopus 900 Haag Streit), electroretinography (ERG) and visual evoked potentials (VEP) (LKC UTAS E3000 LKC Technologies, Inc., United States). Spectral-domain optical coherence tomography (SD-OCT, Cirrus 4000 HD, Carl Zeiss Meditec, Inc., Dublin, CA, United States) was performed to evaluate retinal nerve fiber layer (RNFL) thickness and monitoring disease progression.

**Figure 1:**
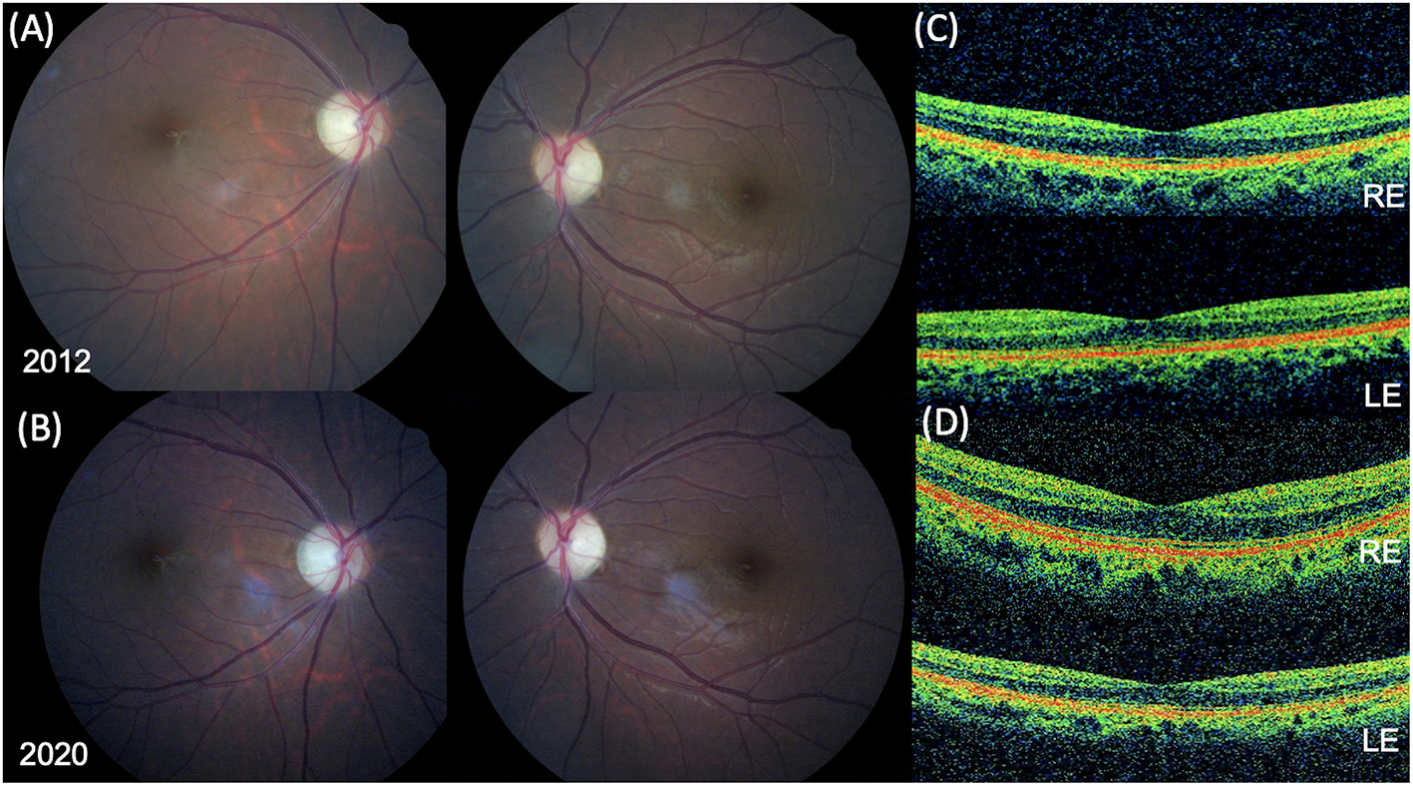
Fundus photographs and spectral-domain optical coherence tomography (SD-OCT) findings. Fundus photograph in 2012 **(A)** and in 2020 **(B)** showing bilateral optic disc temporal pallor with normal macula in both eyes. **(C)** SD-OCT foveal B-scan in 2012 revealing a normal ellipsoid zone (EZ) and a central foveal thickness (CFT) of 246 μm in the right eye (RE) and 239 in left eye (LE). **(D)** SD-OCT foveal B-scan in 2020 confirming a normal EZ and a CFT of 229 μm in the RE and 239 in the LE.

**Figure 2:**
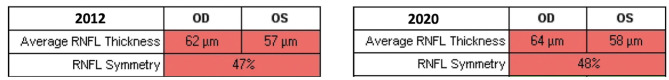
Retinal nerve fiber layer (RNFL) thickness evaluated with spectral domain-optical coherence tomography. Average RNFL thickness and RNFL symmetry did not change significantly throughout the follow-up period.

**Figure 3:**
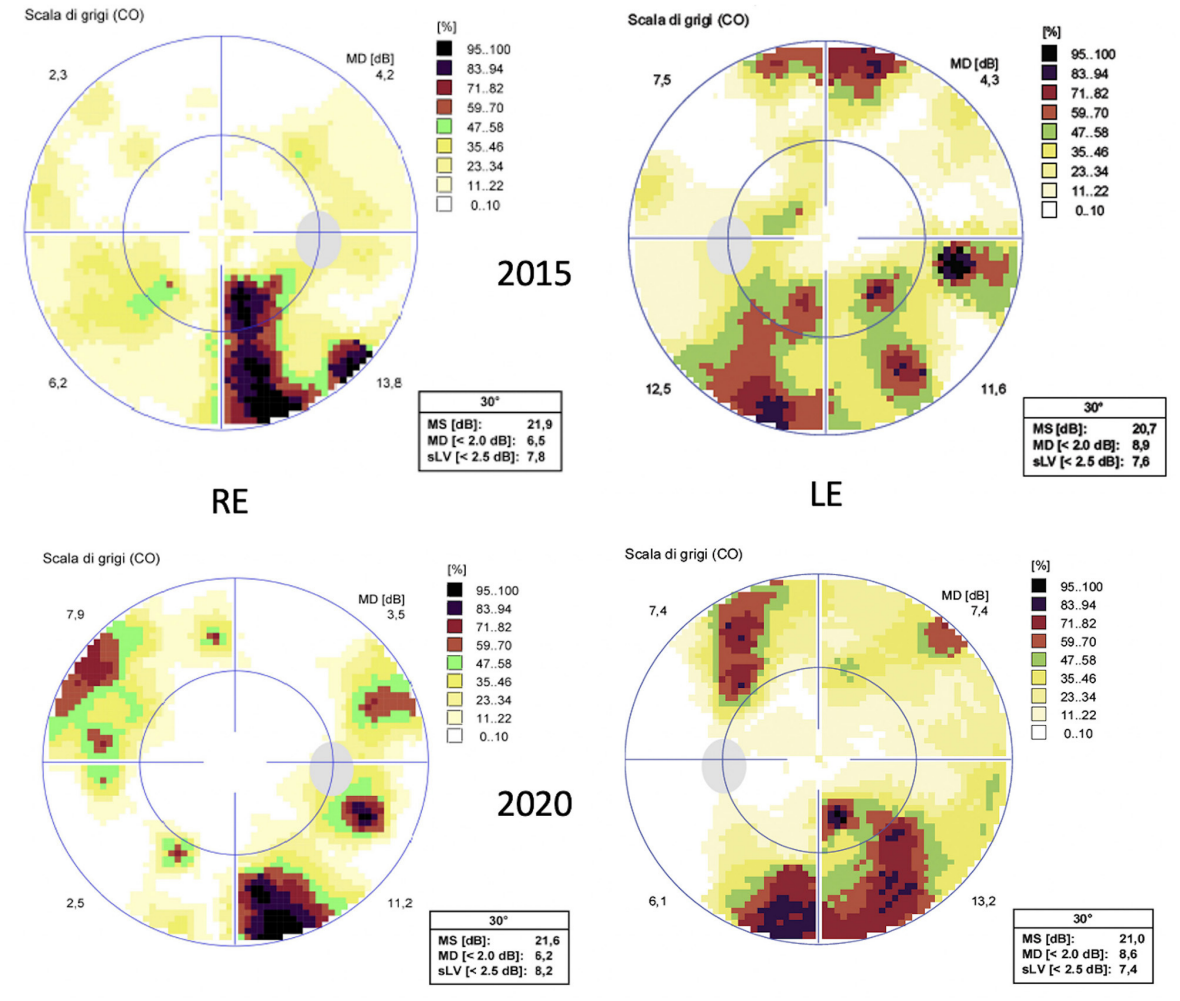
Octopus visual field testing (32 standard). Above the exam performed in 2015 and below the exam performed in 2020, showing stable perimeter indices over the years.

At the last ophthalmic examination in September 2020, patient's BCVA was 20/40 in right eye (RE) and 20/50 in left eye (LE), the same visual acuity presented at the first visit in 2012. Anterior segment slit lamp examination and intraocular pressure (<20 mmHg) were normal bilaterally at each control. Pupils were isochoric, normoreactive to light and no relative afferent pupillary defect was detected. Patient presented no signs of nystagmus, strabismus, and dyschromatopsia on color test evaluation. Fundus examination was stable over time showing bilateral optic disk temporal pallor with normal macula in both eyes and no signs of diabetic retinopathy (Figures 1A,B). Macular and optic nerve SD-OCT measurements were stable after 8 years of follow-up (Figures 1C,D, 2). Central foveal thickness, in 2012 was 246 and 239 μm in RE and LE, respectively. In 2020 it was 229 μm in the RE and 239 μm in LE, with a preservation of the inner and outer retinal layers (Figures 1C,D). Regarding optic nerve analysis, in 2012 SD-OCT showed a reduction of the peripapillary RNFL, a suffering of superior and inferior axonal fibers of optic nerve in both eyes and of nasal axonal fibers in LE. As shown in Figure 2 all these findings were stable during the follow-up. The visual field tests were also stable over the time. Figure 3 shows Octopus visual field with values of a mean deviation of 6.5 dB in RE and 8.9 dB in LE in 2015, and a mean deviation of 6.2 dB in RE and 8.6 dB in LE in 2020. ERG tests were normal in all visits, whereas VEP showed latency increase and amplitude reduction in P100 waves stable throughout the follow-up.

## Discussion

To the best of our knowledge, this is the longest follow-up in the literature in a patient with WFS2 and CISD2 intragenic deletion.

In this report we show that WFS2 can be characterized by an optic neuropathy rather than an OA, with a very slow progression over the time.

To date, few cases of WFS2 with mutations in CISD2 have been reported in addition to our case: 3 consanguineous families of Jordanian descent (6), two Italian siblings (4), a Moroccan patient (9), and a Chinese patient (10). All the aforementioned papers report patients with a severe vision loss developing OA and, in some cases, neurological impairment with progressive cognitive disturbances (11). This variability could be partially due to the different mutations in the CIDS2 gene and to different degrees of penetration. The described phenotype of all cases suggests a heterogeneity of the clinical spectrum, that is: all cases described (with different exon mutations of the CISD2 gene) reported non-autoimmune diabetes, deafness and OA as prevalent clinical features. Among these, OA was absent only in our Caucasian case (7), even after a long follow up; while bleeding intestinal ulcers and defective platelet aggregation were absent in a Moroccan case (9).

Our patient was followed up yearly and the clinical picture including all macular and optic nerve examinations along with the BCVA remained stable over time. In our patient the mutation detected probably led to encode a protein that doesn't cause OA but optic neuropathy.

Our findings further confirm that the involvement of the optic nerve in patients with WFS2 does not always manifest with OA. Additionally, the optic neuropathy progression over the years can be very slow allowing for a stable visual acuity.

Ophthalmological evaluations in WFS2 patients with CISD2 intragenic deletion should be performed longitudinally. All retinal examinations may be helpful in distinguish the patients presenting with OA from those presenting with optic neuropathy, considering the differences in term of prognosis. Indeed, WFS symptoms have a negative impact on individuals' daily function and quality of life. Apart from the finding of a neurogenic bladder, the clinical picture of our patient has been stable over the years, especially as regards the optic neuropathy which allowed her to maintain a good visual acuity and not go blind. She also managed to graduate, due to the stability of the clinical picture over time. The long follow-up of our patient allows us to hypothesize that not all WFS2 cases evolve to blindness. Further studies with comprehensive evaluation of the visual function of patients with WFS2 are needed to clarify the results of our study.

## Data Availability Statement

The original contributions presented in the study are included in the article/supplementary material, further inquiries can be directed to the corresponding author.

## Ethics Statement

Ethical review and approval was not required for the study on human participants in accordance with the local legislation and institutional requirements. The patients/participants provided their written informed consent to participate in this study.

## Author Contributions

VD and EM were responsible for the initial plan and study design. VD, EM, FR, and FD collected and extracted data. VD and EM interpreted data and drafted the manuscript. VD, EM, FR, FD, RG, FT, CI, AF, and FS did a critical revision of the manuscript. All authors contributed to the article and approved the submitted version.

## Conflict of Interest

The authors declare that the research was conducted in the absence of any commercial or financial relationships that could be construed as a potential conflict of interest.
